# Influence of Family Integrated Care on the Intestinal Microbiome of Preterm Infants With Necrotizing Enterocolitis and Enterostomy: A Preliminary Study

**DOI:** 10.3389/fped.2021.678254

**Published:** 2021-11-26

**Authors:** Mengyang Yang, Juan Du, Qin Yang, Wenyan Dou, Min Jiang, Mingyan Hei

**Affiliations:** Department of Neonatology, Neonatal Center, Beijing Children's Hospital, Capital Medical University, National Center of Children's Health, Beijing, China

**Keywords:** intestinal microbiome, family integrated care, preterm infants, necrotizing enterocolitis, enterostomy

## Abstract

The aim of this study was to investigate the influence of family integrated care (FICare) on the intestinal microbiome of preterm infants with necrotizing enterocolitis and enterostomy. This was a prospective pilot study at Beijing Children's Hospital. Premature infants with an enterostomy who met the enrollment criteria were divided into the 2-week FICare and non-FICare groups (non-randomly). We collected their fecal samples and subjected the intestinal microbiomes to 16S rRNA gene sequencing. Operational taxonomic units (OTU) were analyzed to assess the intestinal microbiome richness, and we then carried out α-diversity, β-diversity, and species clustering analyses and a linear discriminant analysis (LDA) effect size (LEfSe) analysis to identify the differences in the microbial communities between the two groups. There were 12 patients enrolled in the study (FICare, *n* = 7; non-FICare, *n* = 5). There were no significant between-group differences in demographic characteristics, or in the relative abundances of phyla and genera. The major bacterial phyla were Proteobacteria, Firmicutes, and Actinobacteria, and *Serratia, Enterococcus, Cronobacter*, and *Bifidobacterium* dominated at the genus level. The α-diversity analysis indicated that the intestinal flora was more diverse in the non-FICare group than the FICare group (*p* < 0.05). However, most of the other indicators did not suggest a difference between the two groups. There was a high proportion of shared OTUs between the two groups, and the PCoA and clustering analyses indicated that the two groups were difficult to distinguish, indicating that the intestinal microbiomes were relatively similar between the groups. In summary, short-term FICare had no significant positive effect on the establishment of intestinal flora diversity in premature infants with necrotizing enterocolitis and enterostomy. The trial was registered in the Chinese Clinical Trial Registry (ChiCTR-OPN-17011801).

## Introduction

The gut microbiota is a major factor influencing both health and disease (1). The composition and development of the microbiota during early life affects health into adulthood (2). Infants who need intensive care are usually nursed in high-sanitary incubators, receive antibiotics, have restricted breastmilk intake, and have limited contact with the mother's skin. These factors all affect the development of the gut microbiota (3). How to improve infants' intestinal flora in the neonatal intensive care unit (NICU) is a current research focus.

From 2014 to 2017, our research group conducted a multicenter prospective cluster-randomized controlled trial on the effect of family integrated care (FICare) on premature infants in China (4), and a study based on our series of FICare trial showed that FICare improved the richness and diversity of the intestinal microbiome in premature infants who didn't undergo surgery in the NICU and helped to establish a microbiome that was similar to that in term infants in the NICU who were breastfed and did not undergo surgery (5). As a novel model of NICU care, FICare allows the infant's parents to participate in the care of the infant in the NICU. A previous study showed that FICare resulted in a 25% increase in weight gain, an 80% increase in the rate of breastfeeding, a 25% decrease in parental stress, and a significant reduction in nosocomial infections and critical incident reports compared to the results in matched controls (6). FICare provides more mother-to-child contact and more breastfeeding opportunities for premature babies in their early lives, which could improve the intestinal microbiome of premature infants in the NICU.

Infants with necrotizing enterocolitis (NEC), spontaneous intestinal perforation, or intestinal atresia commonly require abdominal surgery and the creation of a small bowel enterostomy (7). These children face complex detrimental environmental factors, such as the interruption of the intestine and intraluminal contact with air via a stoma (8), so it is more difficult for normal intestinal flora to become established. There are few studies on intestinal flora issues in infants with enterostomy (9). To investigate the influence of FICare on the intestinal microbiome of newborn infants with NEC and enterostomy in the NICU, we collected fecal samples from neonates who underwent enterostomy owing to NEC, and subjected their intestinal microbiota to high-throughput sequencing.

## Methods

### Ethical Approval

This prospective study was performed at Beijing Children's Hospital from January 2018 to January 2020. The trial was registered in the Chinese Clinical Trial Registry (ChiCTR-OPN-17011801) in 2017. The study was conducted according to the principles of the Declaration of Helsinki and its revisions, and ethical approval was obtained from the Medical Ethics Committee of Beijing Children's Hospital (#2017-106). All parents of the infants provided signed informed consent before participation.

### Infants

**Figure 1:**
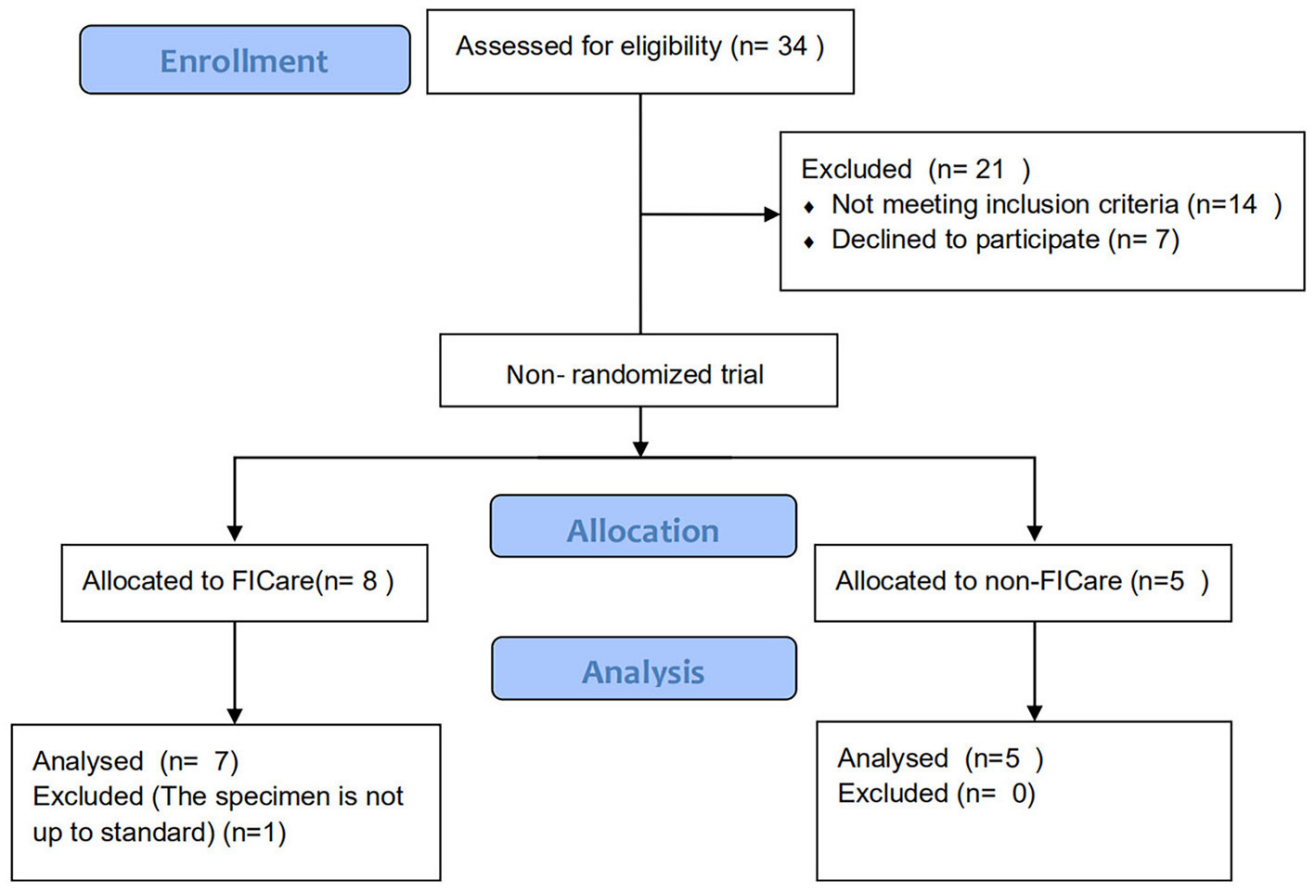
Flowchart of patient enrollment.

Premature infants who were diagnosed with NEC and underwent enterostomy followed by a stay in the NICU at Beijing Children's Hospital were divided into the FICare group (case group) and the non-FICare group (control group). The trial inclusion criteria were as follows: (a) gestational age >28 and <35 weeks; (b) has begun enteral feeding and continuous increase in milk supply for >24 h; (c) stable vital signs (defined as not requiring any ventilation support nor inotropic agent, and the patients' HR/BP/R/SpO2 are in normal range) for >48 h; (d) the first diagnosis was neonatal necrotizing enterocolitis (NEC); and (e) with jejunostomy, ileostomy, or colostomy. The exclusion criteria were as follows: (a) need for invasive ventilation; (b) major congenital anomaly or metabolic disease; (c) family unwilling to commit to staying in the hospital for more than 6 hours per day;(d) lack of consent; (e) birth weight (BW) < 400 g; (f) parental social factors or language barriers, that affect the treatment of the infant; (g) possible discharge within 2 weeks; and (h) oral intestinal probiotics taken after birth. Among the 34 FICare patients with enterostomy, 7 patients were finally included in the FICare group and 5 patients were in the non-FICare group. The detailed enrollment flowchart was in Figure 1. We collected data on the infant characteristics (sex, gestational age, BW, age at the start of enteral feeding, age at NEC diagnosis, and age at enterostomy, rate of breastfeeding, duration of supplemental oxygen use, duration of antibiotic use, top three diagnoses except NEC, weight at discharge, and 5-min Apgar score) and maternal characteristics (age, antenatal steroid use, prolonged rupture of membranes >18 h, delivery mode, and major maternal medical complications) (Table 1).

**Table 1:** Baseline data: Infant demographics and maternal characteristics.

**Characteristic**	**FICare (7)**	**Non-FICare (5)**	***p*** **value**
Male sex, *n* (%)	4 (57)	3 (60)	0.56
Gestational age, week, median (IQR)	31 (29.6–31.6)	32 (29.7–32.3)	0.45
Birth weight, g, mean (SD)	1510 (448.8)	1614 (502.2)	0.72
Singleton, n (%)	3 (43)	3 (60)	1
Cesarean delivery, *n* (%)	3 (43)	4 (80)	0.29
Apgar <7 at 5 min, *n* (%)	1 (14.3)	1 (20)	1
Prolonged rupture of membranes >18 h, n (%)	1 (14.3)	2 (40)	0.52
Maternal age, year, mean (SD)	30.3 (4.8)	29.2 (4.2)	0.69
Complete antenatal corticosteroids, *n* (%)	2 (29)	2 (40)	1
Breastfeeding, *n* (%)	6 (86)	2 (40)	0.20
Maternal complications, *n* (%)			
Hypertension	2 (29)	0	0.03
Gestational diabetes	0	1 (20)	0.42
Thyroid dysfunction	0	0	
(Suspected) sepsis	0	2	0.15
Main diagnosis of infants (except NEC and prematurity), *n* (%)			
Respiratory distress syndrome	7 (100)	5 (100)	-
Perinatal asphyxia	3 (43)	1 (20)	0.58
(Suspected) sepsis	0	1 (20)	0.42
Hypoglycemia	0	0	
Pneumonia	2 (29)	1 (20)	1
Age at the start of enteral feeding, d, median(IQR)	2 (2, 4)	2 (2, 8)	0.39
Age at NEC diagnosis, d, mean (SD)	14 (4)	12.6 (8.8)	0.72
Age at enterostomy, d, mean (SD)	13.3 (6.5)	17 (8.2)	0.40
Duration of supplemental oxygen, d, mean (SD)	28.4 (19.5)	17.8 (11.4)	0.30
Duration of antibiotic use, d, mean (SD)	17.6 (6.8)	10.8 (3.6)	0.07
Weight at discharge, g, mean (SD)	2155 (88.5)	2295 (178.6)	0.10

### FICare Implementation and Sample Collection

After the enterostomy surgery was finished, the newborns were admitted to the NICU. After their vital signs stabilized and antibiotics had been finished for 1 week, they were divided into the two groups. We had established a professional FICare team and transformed the wards accordingly. Only the mothers of FICare newborns were allowed to enter the NICU for direct infant care, but the education and coaching sessions were open to both parents. Site training included bundles of written protocols, printed parent education materials, printed staff training materials, an onsite FICare training workshop and hands-on training, and an all-site training meeting in Beijing. A total of 13 non-invasive care skills were taught to the parents by the FICare team, which included the six-step washing technique, positioning of newborn babies, diaper use and estimation of urine output, umbilical cord care, oral care, and kangaroo skin contact. Our team also guided the parents on recording the baby's general condition, body temperature, heart rate, weight, urine output, milk intake, vitality and mood. The NICU team provided practical support and the FICare implementation policy details (10). The FICare newborns were given FICare for 2 weeks. The parents in the non-FICare group did not receive the FICare training and their infants were provided with standard care.

After 2 weeks of FICare, fresh feces were collected from the infants in the FICare group over the following week (≥2 times, with intervals >24 h). Non-FICare group samples were collected at corresponding time points. A total of 38 samples were collected from the 12 premature NEC infants with enterostomy. Each tube containing a fecal sample was sealed with a sealing membrane and rapidly stored in a −80°C freezer for later analysis. All fecal samples were sent to a testing company for 16S rRNA high-throughput sequencing of the intestinal flora.

### Data Processing and Analysis

After extracting the total DNA from the samples, we amplified the V4 region of the 16S rRNA gene by using polymerase chain reaction (PCR) with a 515F (5′-GTGCCAGCMGCCGCGGTAA-3′) and 806R (5′-GGACTACHVGGGTWTCTAAT-3′) primer pair, which had sequencing adapters attached to the ends. After amplification, the PCR products were subjected to purification, quantification, and homogenization to construct the sequencing library. Purified amplicons were pooled using equimolar concentrations and paired-end sequenced (2 × 251 bp) on an Illumina MiSeq platform (Illumina, San Diego, CA, USA)

MiSeq Reporter software v2.5.1.3 (Illumina, San Diego, CA, USA) with default parameters was used to split the raw sequencing data according to each sample's sequences, and to remove the sub and low-quality sequences. We obtained the V4 region assembly sequences after quality control and sequence splicing. i.e., the raw tags. To obtain high-quality tags, tags <225 or >280 bp were filtered out and chimeric sequences were removed using UCHIME v4.2 (11) (http://drive5.com/uchime).

After the Tags were optimized, an operational taxonomic unit (OTU) analysis was used to cluster the sequences at the 97% similarity level using UPARSE (singleton OTUs with an abundance of 1 in a single sample, which may represent very rare OTUs or may be caused by sequencing errors, were filtered out) (12). The species were annotated and a taxonomic analysis was carried out using the Silva 16S rRNA gene database, employing a confidence threshold of 80% (13). We used a Venn diagram to visualize the number of shared and unique OTUs in the two groups. Thereafter, based on the representative sequences in each OTU, α-diversity, β-diversity, and species clustering analyses were carried out. Regarding the α-diversity analysis, the species abundance indices (ACE and Chao), Simpson index, and Shannon index were used to evaluate the species abundances and diversity of the microbial communities. Regarding the β-diversity analysis, species diversity between samples was calculated using weighted and unweighted UniFrac approaches. Mothur software was used to conduct these analyses (14). Moreover, principal coordinate analysis (PCoA) results were plotted using the PCA command in Mothur, which was used to perform principal component analysis of the microbial flora in each sample to assess the similarity between the samples. Correlation analysis results were visualized using heatmaps. Furthermore, linear discriminant analysis (LDA) effect size (LEfSe) analysis (also known as biomarker analysis), which analyzes significant differences between groups, was used to estimate the impact of the abundance of each component (i.e., taxonomic group) on the difference between the two groups (15).

### Statistical Analysis

Continuous data with normal distribution, we presented the data with mean (SD), and for data with non-normal distribution, we presented the data with median (IQR) are expressed as mean ± standard deviation (SD). If the data were normally distributed, the two groups were compared using the independent-samples *t*-test. If the data were not normally distributed, the two groups were compared using the Mann–Whitney U test. Categorical data are expressed as frequency (%), and the chi-square test was used to compare the two groups. *P* < 0.05 was considered statistically significant.

## Results

### Participants

There were 12 infants who met our eligibility criteria and were enrolled in the study, comprising 7 in the FICare group and 5 in the non-FICare group. The demographic and clinical information on the newborns in the two groups is shown in Table 1. The majority of premature infants included in this study were born in our hospital, with a mean gestational age of about 30 weeks and a mean BW of about 1,500 g. There were no significant differences in demographic or clinical characteristics such as the proportion of male infants and the proportion with Apgar score <7 at 5 min. There was a significant difference in maternal hypertension, but not in the other complications. The age at NEC diagnosis was about 12 days for both groups (12 ± 5.3 vs. 12.6 ± 8.8, *P* = 0.72). In addition to the diagnosis of NEC, all the FICare and non-FICare preterm infants had a diagnosis of respiratory distress syndrome, while 43 and 20% of the FICare and non-FICare groups, respectively, had perinatal asphyxia (*p* = 0.576). The FICare group had a longer duration of supplemental oxygen use than the non-FICare group (28.4 ± 19.5 vs. 17.8 ± 11.4), a longer duration of antibiotic use (17.6 ± 6.8 vs. 10.8 ± 3.6), a younger age at enterostomy (15.3 ± 4.9 vs. 17 ± 8.2), and a higher breastfeeding rate (86 vs. 40%), but none of the differences were significant.

### Relative Composition analysis

We collected 20 samples from the FICare group and 18 samples from the non-FICare group. Among them, 3 samples in the FICare group did not meet the required standards and were discarded. After processing, a total of 1,571,424 high-quality Tags were obtained from the 35 samples, with a mean of 44,897 per sample.

**Figure 2:**
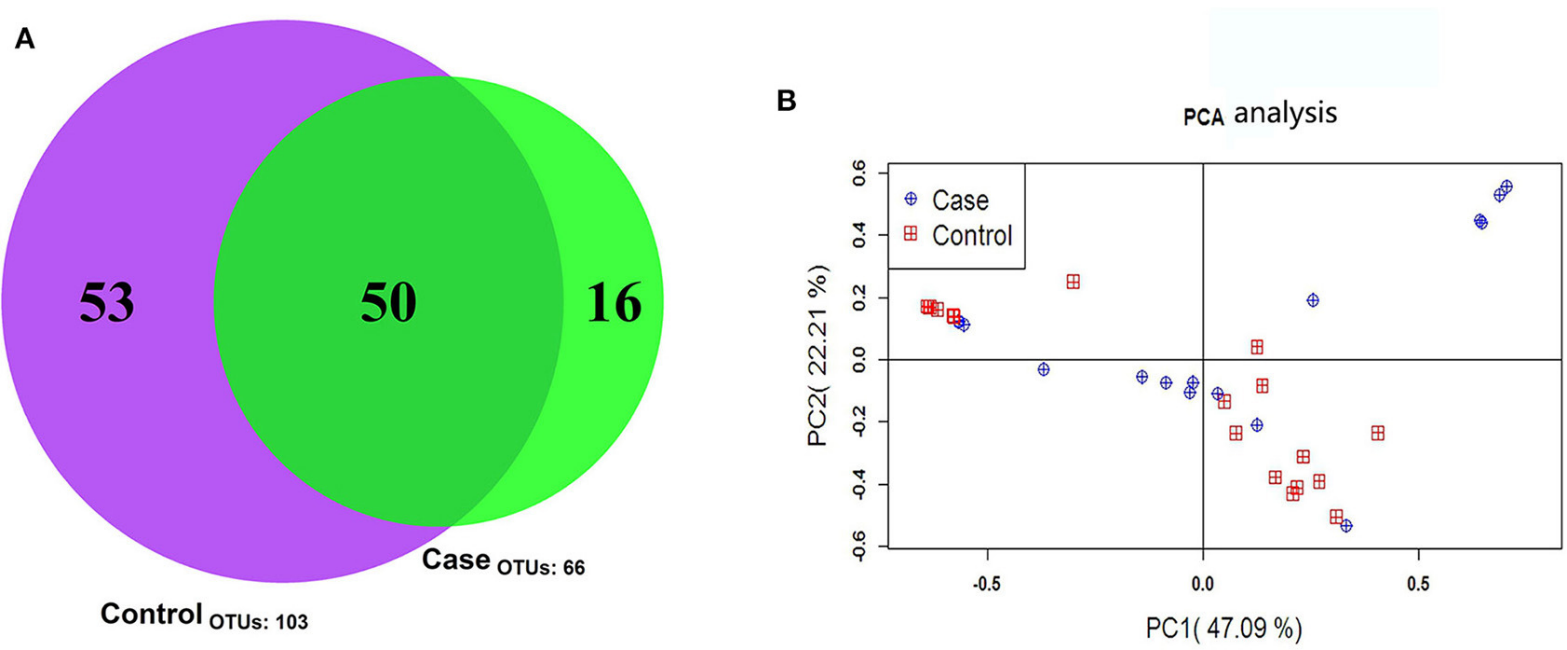
**(A)** OTU Venn diagram **(B)** principal component analysis score plots of fecal microbial composition within the case and control group. The first and second component are shown on the x- and y-axis.

The 35 samples produced 119 OTUs. The minimum number of high-quality Tags in these samples was 11,347. The shared and distinct OTUs in the two groups were plotted in a Venn diagram (Figure 2A). The 50 OTUs were shared by the two groups The FICare group had 16 unique OTUs, while the non-FICare group had 53 unique OTUs. The abundance of OTUs preliminarily indicated the species richness of the samples. The results showed that the richness and diversity of intestinal flora was higher in the non-FICare group than the FICare group, but there were many shared OTUs in the groups.

PCoA is a flexible tool for analyzing microbiota composition data (16) that allows the overall effects of different treatments or environments on microbiota composition to be assessed. In PCoA plots, the dispersion or aggregation can reflect the differences in the gut microbiota in the two groups being compared based on the OTU composition (97% similarity) of the various samples. The results revealed no differences in microbiota composition between the two groups (Figure 2B).

**Figure 3:**
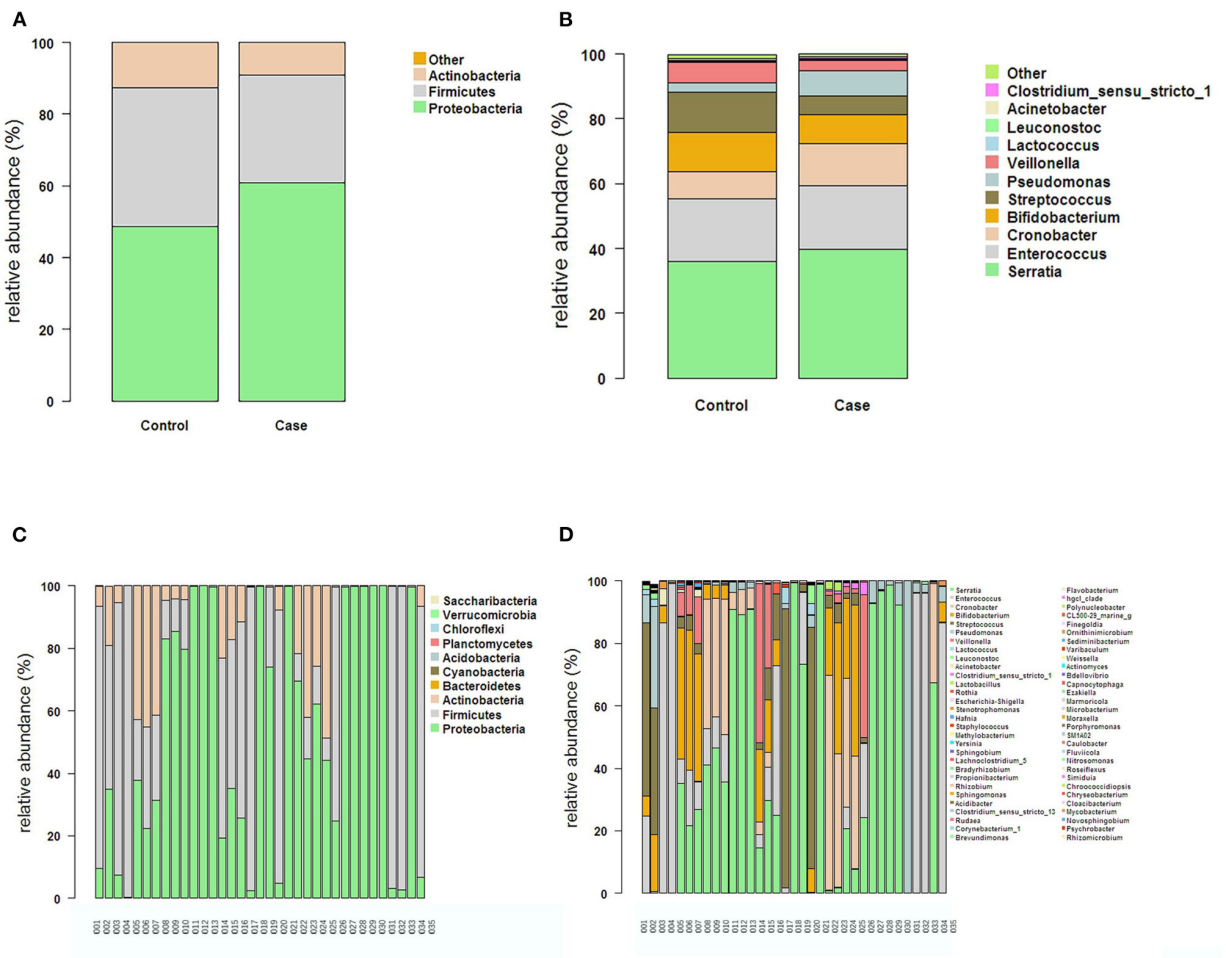
The structure and relative abundance of the flora in the two groups at the Phylum level **(A)** and the genus level **(B)**. The structure and relative abundance in each sample at the phylum level **(C)** and the genus level **(D)**.

The intestinal flora structure and relative bacterial abundances in the fecal samples are shown at the phylum level (Figures 3A,C) and genus level (Figures 3B,D). At the phylum level, the major bacteria in both groups were Proteobacteria, Firmicutes, and Actinobacteria. The most abundant phylum in both groups was Proteobacteria. There were no significant differences in relative abundance between the two groups at phylum level (*p* > 0.05) (Table 2). The proportion of Proteobacteria was higher in the FICare group (60.9 ± 9.68%) than the non-FICare group (48.41 ± 8.97%), and Proteobacteria and Firmicutes also accounted a higher proportion in the FICare group (90.9%) than in the non-FICare group (87.4%). The mean number of intestinal bacterial genera per infant in the FICare and non-FICare group was 13.6 ± 6.7 and 11.6 ± 5.9, respectively, but the difference was not significant (*p* = 0.35). At the genus level, *Serratia, Enterococcus, Cronobacter*, and *Bifidobacterium* were dominant, accounting for >90% of the total. There were no significant differences in relative abundance at the genus level (*p* > 0.05) (Table 3). The proportion of *Bifidobacterium* was higher in the non-FICare group than the FICare group (5.05 vs. 0.05%), but the difference was not significant (*p* = 0.195).

**Table 2:** Proportions of intestinal flora phyla in fecal samples in the two groups.

**Group**	**Proteobacteria, mean ± SD**	**Firmicutes, mean ± SD**	**Actinobacteria, mean ± SD**
FICare	60.92 ± 9.67	30.02 ± 9.65	9.06 ± 3.8
Non-FICare	48.57 ± 8.95	38.86 ± 8.30	12.54 ± 3.71
p value	0.33	0.46	0.53

**Table 3:** Proportions of intestinal flora genera in fecal samples in the two groups.

**Group**	***Serratia***, **mean ± SD**	***Enterococcus***, **mean ± SD**	***Cronobacter***, **mean ± SD**	***Bifidobacterium*** ^*^	***Pseudomonas*** ^*^	***Streptococcus*** ^*^	***Veillonella*** ^*^
FICare (7)	39.71 ± 10.52	19.61 ± 8.69	13.02 ± 5.31	0.05 (0,14.61)	1.3 (0.095,4.415)	0.24 (0,2.985)	0.01 (0.005,1.35)
Non -FICare (5)	35.91 ± 8.16	19.28 ± 6.90	8.43 ± 3.59	5.05 (0.025,19.5825)	0.28 (0.0175,2.0375)	1.215 (0.1,11.215)	0.005 (0,8.0925)
*p* value	0.777	0.976	0.474	0.195	0.134	0.134	0.443

* *Expressed as median (lower quartile, upper quartile) and analyzed using non-parametric tests*.

After assessing the between-group differences in microbial abundances using statistical tests, a false discovery rate (FDR) analysis was used to further evaluate the significance of the differences, so as to identify the bacteria underlying the composition differences between the two groups. We attempted to identify the significant differences between the groups at both the phylum and genus levels. No significant differences were found between the two groups at the phylum level. At the genus level, there were 7 genera with significant differences in abundance between the two groups based on the p values (*p* < 0.05) (Table 4). Based on the FDR analysis, four bacteria were found to exhibit significant between-group differences in the proportions based on the q values (q < 0.05), comprising *Clostridium_sensu_stricto_13, Rudaea, Hafnia*, and *Clostridium_sensu_stricto_1*. However, the proportions of these four bacteria in the two groups were all <1%.

**Table 4:** False discovery rate (FDR) analysis of differences in abundances between the two groups.

**Genus**	**Non-FICare**		**FICare**		***p*** **value**	***q*** **value**
	**Mean**	**SD**	**Mean**	**SD**		
*Clostridium_*	0.000069	0.000048	0	0	0.000131	0.003825
*sensu_stricto_*						
*13*						
*Rudaea*	0.000069	0.000069	0	0	0.000131	0.003825
*Hafnia*	0.001783	0.000987	0	0	0.002997	0.043758
*Clostridium_*	0	0	0.005179	0.002587	0.002997	0.043758
*sensu_stricto_1*						
*Yersinia*	0.000304	0.000154	0.000005	0.000005	0.004995	0.058344
*Acinetobacter*	0.005427	0.00308	0.000223	0.000109	0.007992	0.077792
*Staphylococcus*	0.001439	0.000565	0.000135	0.000098	0.011988	0.100019

### LEfSe Analysis

**Figure 4:**
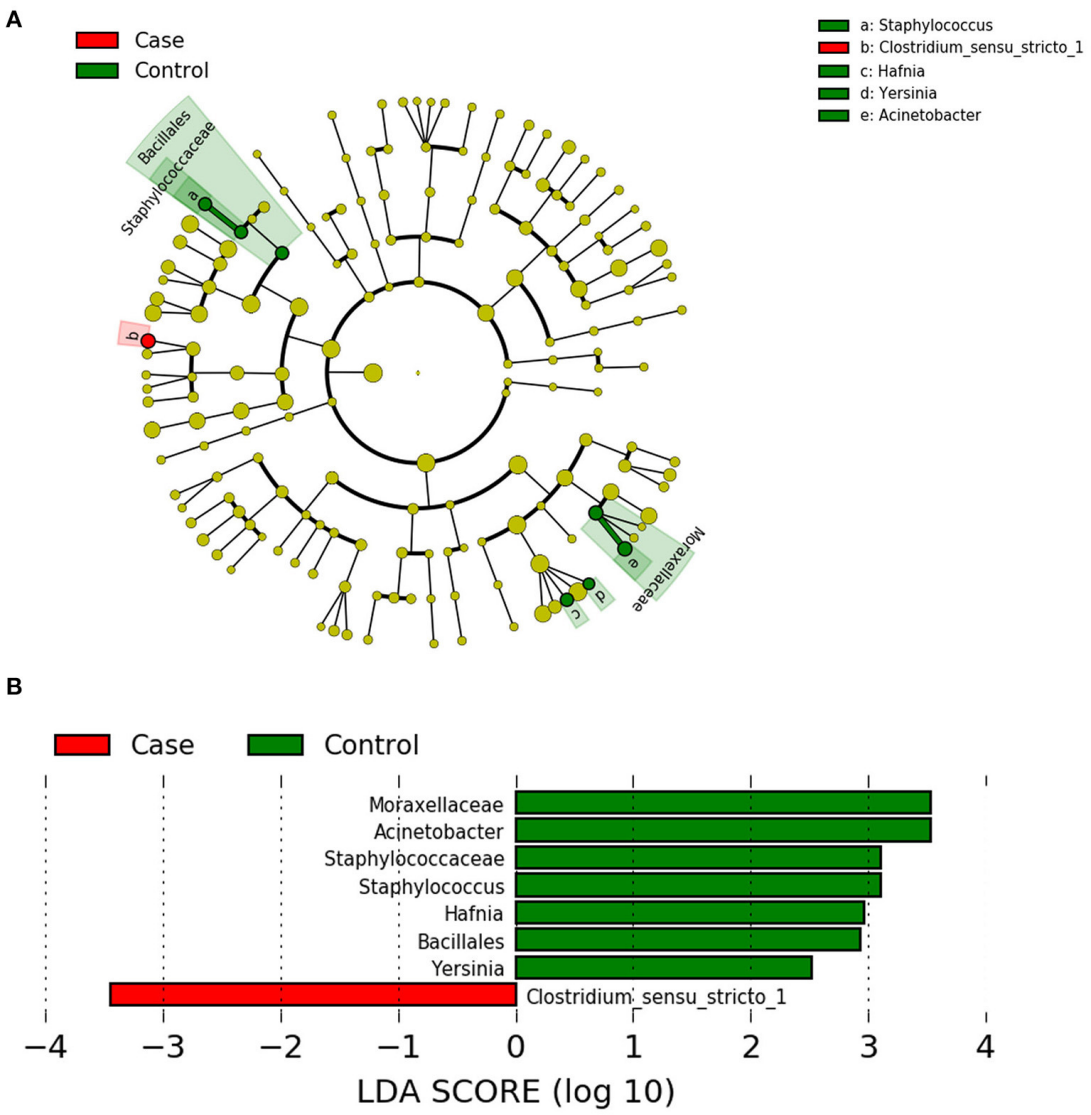
**(A)** LEfSe cladogram, the diameter of each circle is proportional to the abundance. **(B)** Histogram of the LDA scores for diferentially abundant features among groups.

LEfSe analysis can be used to demonstrate different gut microbiota structures between two groups and aid in identifying significantly differential biomarkers between groups (15). LEfSe uses LDA to estimate the influence of the abundance of each component (i.e., taxonomic group) on the difference between groups. The figure generated in the LEfSe analysis (Figure 4A) shows the taxonomic groups with the largest differences between the two groups at various levels. The histogram (Figure 4B) shows the differences in 8 phylotypes between the two groups. At the genus level, the LEfSe analysis (Table 5) showed that 4 bacteria could be used as biomarkers to distinguish the two groups. In the FICare group, Clostridia (class level) could be used as a biomarker, which is consistent with the FDR analysis results (*Clostridium_sensu_stricto_1* belongs to Clostridia). While in the non-FICare group, *Enterobacteriaceae, Moraxellaceae*, and *Staphylococcaceae* (different families) could be used as biomarkers. Which has two more bacteria than the FDR analysis (*Hafnia* belongs to *Enterobacteriaceae*).

**Table 5:** LDA effect sizes.

**Feature**	**Logarithm value**	**Group**	**LDA score**	***p*** **value**
Bacteria.Firmicutes.Bacilli.Bacillales	3.2233	Non-FICare	2.9327	0.0283
Bacteria.Firmicutes.Bacilli.Bacillales.Staphylococcaceae	3.1580	Non-FICare	3.1052	0.0088
Bacteria.Firmicutes.Bacilli.Bacillales.Staphylococcaceae.Staphylococcus	3.1580	Non-FICare	3.1033	0.0088
Bacteria.Firmicutes.Clostridia.Clostridiales.Clostridiaceae_1.Clostridium_sensu_stricto_1	3.7140	FICare	3.4468	0.0067
Bacteria.Proteobacteria.Gammaproteobacteria.Enterobacteriales.Enterobacteriaceae.Hafnia	3.2512	Non-FICare	2.9684	0.0023
Bacteria.Proteobacteria.Gammaproteobacteria.Enterobacteriales.Enterobacteriaceae.Yersinia	2.4929	Non-FICare	2.5225	0.0077
Bacteria.Proteobacteria.Gammaproteobacteria.Pseudomonadales.Moraxellaceae	3.7364	Non-FICare	3.5304	0.0229
Bacteria.Proteobacteria.Gammaproteobacteria.Pseudomonadales.Moraxellaceae.Acinetobacter	3.7351	Non-FICare	3.5293	0.0239

### Species Clustering Analysis

**Figure 5:**
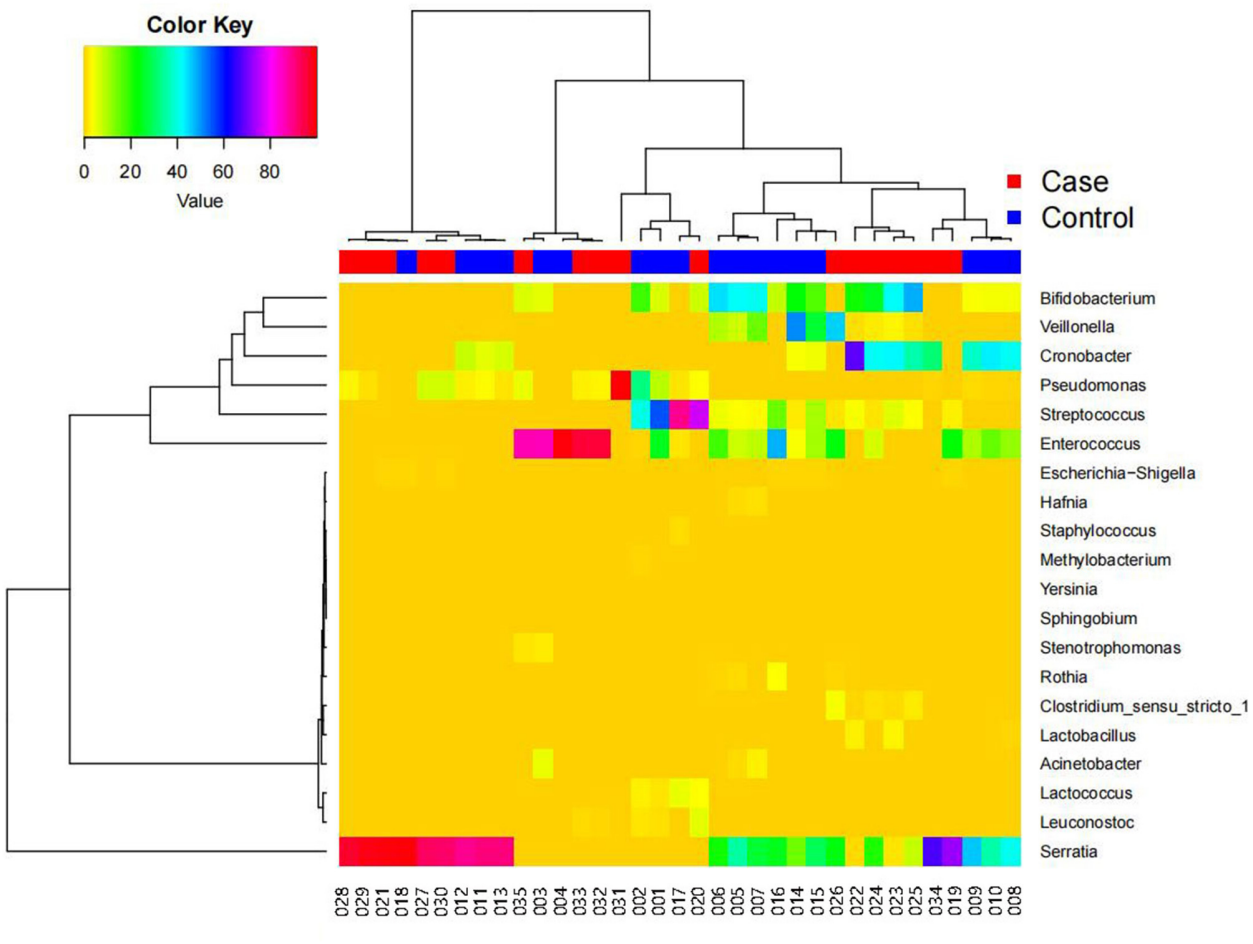
The heat map between bacterial community in two groups at the genus level.

Figure 5 shows a heatmap of the species clustering analysis results at the genus level (top 20 species), which reflects the similarities and differences in community composition among the samples. In the dendrogram above the heatmap, the shorter the branch length, the more similar the species composition and abundance between samples. The heatmap indicates the species abundances in the various samples and the similarities in species-specific abundances in the various samples. There was little difference between the FICare and non-FICare groups, but there were differences to a certain extent in the composition within the FICare and non-FICare group, respectively.

### β-Diversity Analysis

The β-diversity of the gut microbiota in the two groups was assessed, which involved comparing species diversity between each pair of samples. UniFrac uses phylogenetic information to compare community differences between samples, which takes into account the evolutionary distance between species, the difference is represented by the 0-1 distance value, the larger the value, the greater the difference between samples; samples from the same environment are more likely to concentrate at the same node in the evolutionary tree, that is, the branch length between them is short and the similarity is high, so the results can be interpreted as an index of β-diversity.

**Figure 6:**
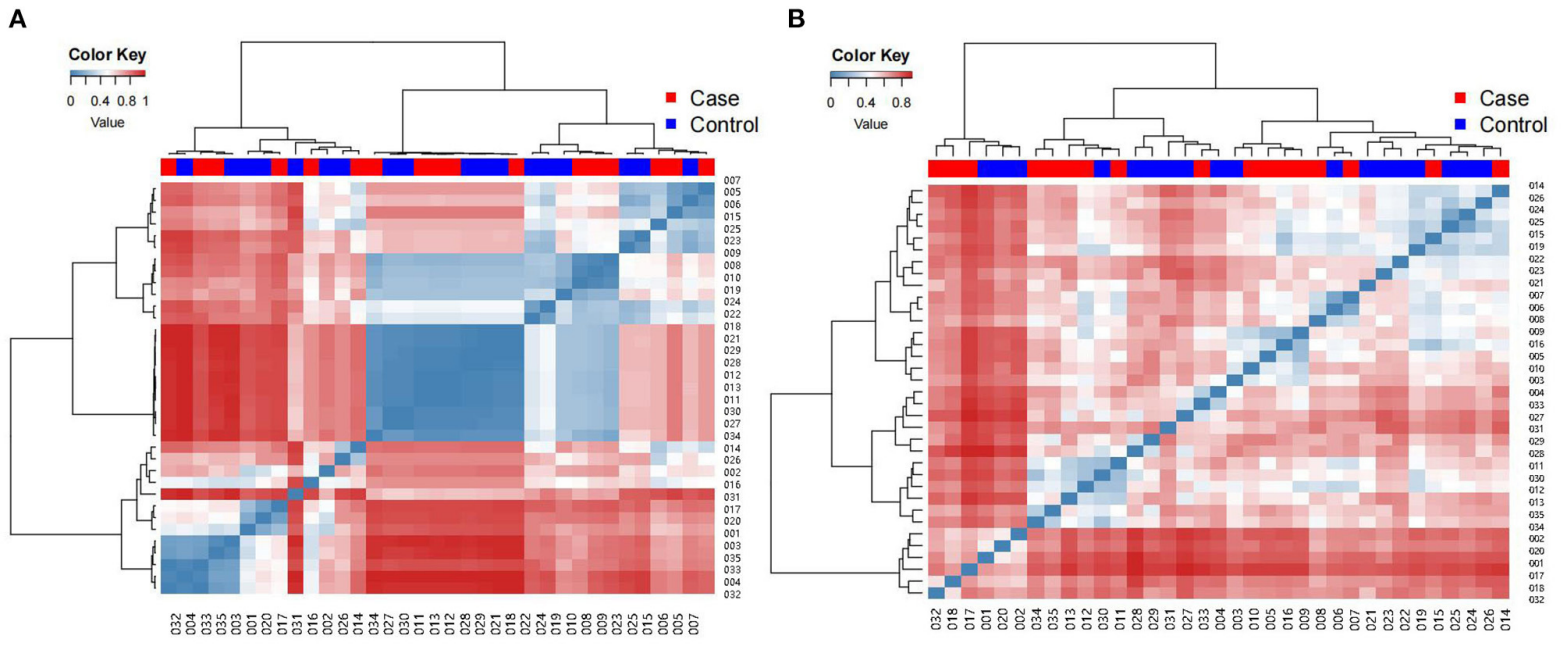
Beta diversity heat map **(A)** is weighted, **(B)** is unweighted.

The clustered UniFrac results (Figure 6), included weighted UniFrac, which takes into account sequence abundance) and unweighted UniFrac results, which only considers whether the sequence is present in the community, not the abundance of the sequence. In the unweighted UniFrac heatmap, many values were close to 1, which indicates that there was a certain difference between the pairs of samples. There may be great differences in microbial community structure between different samples from the same individual. In the weighted UniFrac heatmap, there were many lower values, the results revealed that species similarity was high between the pairs of samples.

**Figure 7:**
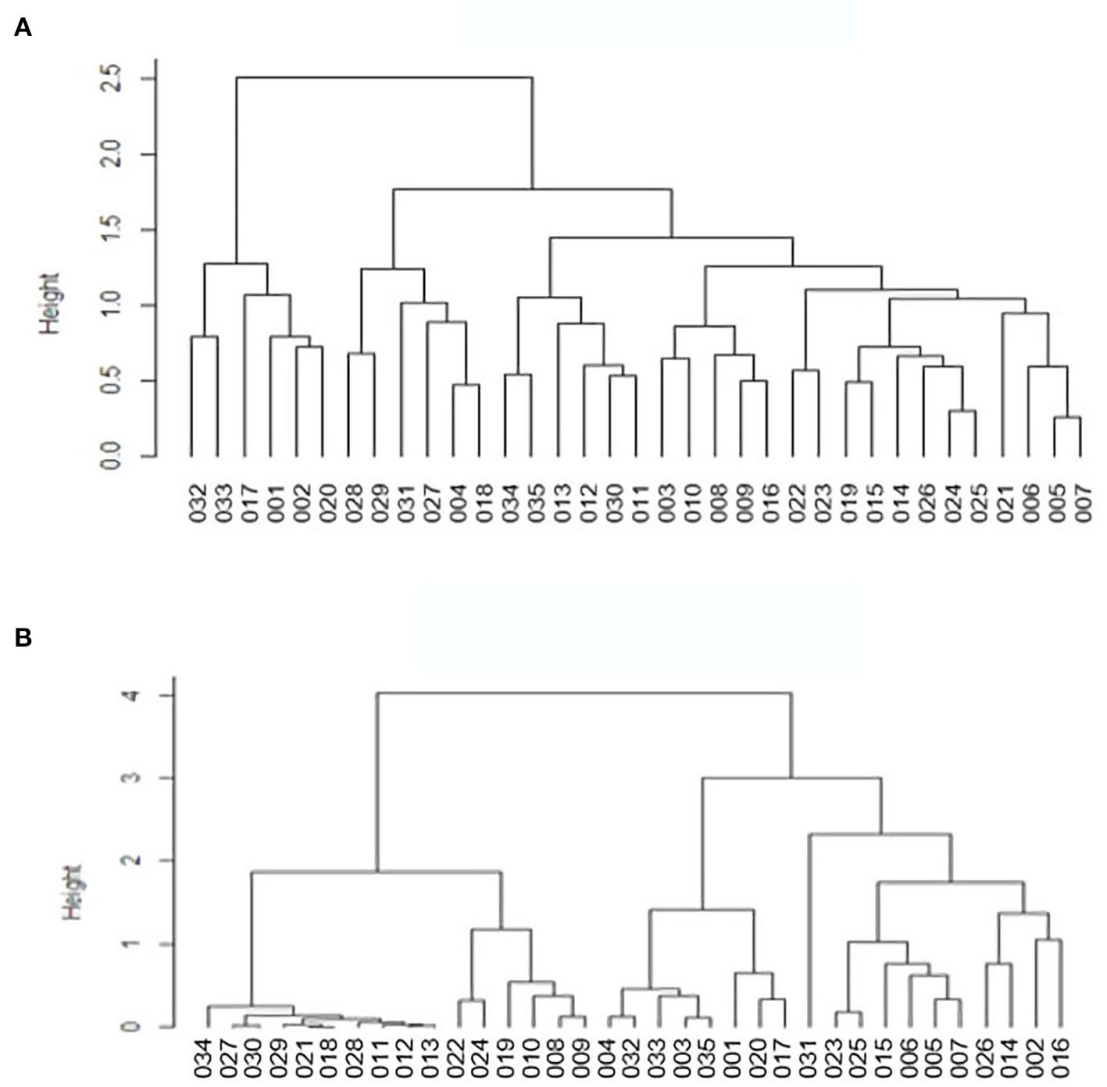
Cluster analysis diagram **(A)** is unweighted, **(B)** is weighted.

Based on the β-diversity results, we carried out dendrogram clustering analyses and calculated the distances between the samples to assess the similarity of species composition. The results are shown in Figure 7. The shorter the branch length in the dendrogram, the closer the samples, indicating similar species composition. The results confirmed that the two groups were similar with small differences.

### α-Diversity Analysis

**Figure 8:**
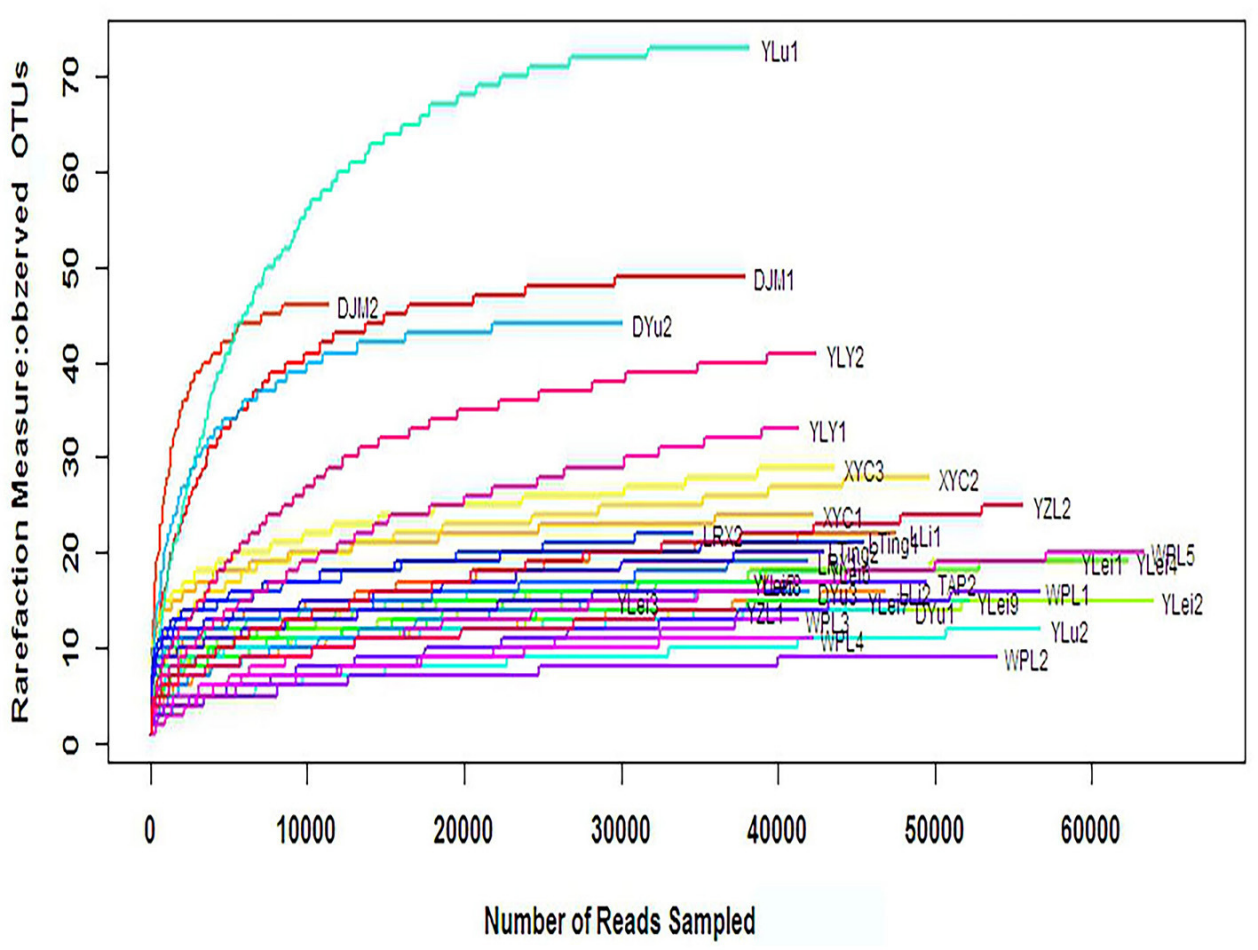
The rarefaction curve of samples.

The α-diversity can reflect the species diversity within a single group. Observed OTUs, Chao, and abundance-based coverage estimator (ACE), which reflect species richness, did not significantly differ between groups (Table 6). The rarefaction curves, with distinct asymptotes, are presented in Figure 8, which indicates near-complete sampling of the microbial communities. Good's coverage was 99.96 ± 0.02 for the observed OTUs.

**Table 6:** Alpha diversity statistical results.

**Index**	**Non-FICare, mean ± SD**	**FICare, mean ± SD**	***p*** **value (KW)**
Good's coverage	0.9997	0.9996	-
Observed OTUs	18.94 ± 13.07	14.82 ± 8.62	0.305
Chao	21.79 ± 14.52	20.78 ± 13.52	0.882
ACE	26.60 ± 15.24	22.92 ± 12.17	0.843
Simpson	0.52 ± 0.28	0.70 ± 0.26	0.045
Shannon	0.99 ± 0.57	0.62 ± 0.20	0.045

Shannon index and Simpson index reflect the diversity of microorganisms in each sample, which is affected by species richness and evenness. The Shannon index is more sensitive to the richness of the community and the rare OTU, and is more suitable for complex communities; while the Simpson index is more sensitive to the uniformity and the dominant OTU in the community, and is more suitable for simple communities. Shannon index in the non-FICare group was significantly higher than that in the FICare group (0.99 ± 0.57 vs. 0.62 ± 0.20, P =0.045) (Table 6), indicating that species richness in the non-FICare group was higher and also consistent with LEfSe results. Simpson index was higher in the FICare group (0.52 ± 0.28 vs. 0.70 ± 0.26, *P* =0.045) (Table 6). Both of them indicated a significantly greater diversity in the non-FICare group.

## Discussion

In this prospective study on the influence of 2 weeks of FICare on the intestinal flora of preterm NEC infants with enterostomy, there was a significant difference in α-diversity between the FICare and non-FICare groups, with higher α-diversity in the non-FICare group. The other indicators did not exhibit significant differences between the two groups. There was a high proportion of shared OTUs in the two groups, and the PCA and clustering analyses could not significantly distinguish the two groups. These findings might have indicated that the intestinal microbes of the newborns in the two groups were relatively similar. However, though the differences were not statistically significant, they could be clinically relevant and influence the outcomes, because the sample size was too small.

FICare (17) is considered a highly comprehensive parent-involved care model for NICU care, and it can promote parental empowerment, learning, shared decision making, and positive parent–infant caregiving experiences. Emerging evidence (18–20) indicates that FICare is a promising intervention for infants and families that could improve outcomes for preterm infants and provide effective parental support. Our previous multicenter prospective cluster-randomized controlled trial demonstrated that FICare (4) is feasible in China and positively influenced the duration of hospitalization, cost of care, duration of supplemental oxygen use, infection, antibiotic use, infant growth, and breastfeeding rate. More research on the effectiveness of FICare is currently underway (21, 22). However, there have been few studies on the care of infants undergoing surgery (23) and there has been no research on whether FICare can improve the gut microbiota of these infants. One study found that two-weeks' FICare can improve the intestinal flora diversity of non-surgical preterm NICU infants (5). Unlike that study, our study indicates that short-term FICare did not exhibit clinical efficacy regarding the intestinal flora diversity for preterm NEC infants with enterostomy in the NICU. The reasons may include the long time required for intestinal flora establishment in premature neonates (24) and the more severe disease that newborns undergoing surgery often have, which necessitates more time for gut flora restoration, so changes may take longer than the 2 weeks in our study. There remains a lack of knowledge about optimum care for preterm infants with enterostomy. As the enrolled neonates had severe health conditions, which often changed rapidly, they required more high-quality care to protect their fragile intestinal flora. Inexperienced parents usually cannot rapidly identify these changes, so they require longer training and education in order to provide appropriate care. This may have contributed to the slightly better diversity in the non-FICare group than in the FICare group. Nevertheless, most infants undergoing surgery also need long-term out-of-hospital care, and it is better for their parents to adapt to the care role early. FICare allowed close contact between the mother and infant, and provided time and space for the mother to breastfeed the neonate in the NICU. The influence of moving from the NICU to home on the intestinal flora can be reduced by allowing long-term mother-to-child contact in the NICU. Due to changes in the environment, handling, feeding, and treatment regimens, the change in the care model, could also bring about multiple effects that could enhance the microbial transmission to neonates. How these factors affect the development of the intestinal flora needs further research. Moreover, we should also pay attention to the longer-term health management of these infants after discharge. Long-term follow-up and investigation of other types of indicators (feeding, growth and development, defecation, and other diseases) and the detailed changes in the intestinal flora are needed to fully assess the effectiveness of FICare. Based on the effects of FICare demonstrated in our previous research (4, 5), it is worthwhile to further research whether FICare is beneficial.

In healthy neonates or preterm infants with mild disease, the intestinal flora is dominated by the genus *Lactobacillus* in the phylum *Firmicutes* (25). Research on mouse models of acute traumatic brain injury indicated that *Bifidobacterium* and *Lactobacillus* improved the electrophysiological peristalsis function of small intestinal smooth muscle during acute traumatic brain injury via the PKC/MLCK/MLC signaling pathway (26), which improved intestinal function. However, data on the extent to which surgical interruption of the intestine affects the developing intestinal microbiota are rare (9). In our study of NEC infants with enterostomy, the intestinal flora was dominated by Proteobacteria, which is one of the biggest bacterial phyla and comprises Gram-negative bacilli, including many pathogenic bacteria. At the genus level, *Serratia, Enterococcus*, and *Cronobacter* accounted for the majority of the bacteria. This is partly consistent with a study by Younge et al. (27), who observed a relative increase in many genera in the Enterobacteriaceae family (including *Serratia, Escherichia, Pantoea*, and *Citrobacter*) in infants with enterostomy. Bacteria in this family can induce potent host inflammatory responses and are frequent invasive pathogens in premature infants and infants with short bowel syndrome (28–30). Several studies have reported that the high level of oxygen in the newborn gastrointestinal (GI) tract favors facultative anaerobes (e.g., *Enterobacteriaceae, Enterococcus*, and *Streptococcus*) (31, 32). Theoretically, the basic conditions of the infants, antibiotic use, and the surrounding environment may underlie any differences in the neonatal intestinal flora between the FICare and non-FICare groups. The causality and effect size of various factors need to be assessed in the future. The enrolled newborns may need a longer time to develop normal intestinal flora. Whether oral probiotics, such as *Bifidobacterium* and *Lactobacillus*, can improve their intestinal flora also needs more research.

The intestinal microbiome has an essential role in intestinal function, including nutrient absorption, metabolism, maintenance of barrier integrity, and protection against infection (33, 34). Caporaso et al. (35) found that the human microbiome exhibited a phenomenon known as adaptation, involving changes in response to environmental factors (such as diet, intestinal transit time, and drug use) followed by changes back to the original composition. Research on laboratory rats has shown that the living environment has a profound effect on the development of the immune system and changed the nature and distribution of immune cells (36). Early establishment of normal intestinal flora is needed for every infant, but preterm infants, who have an immature self-adjustment ability, are more easily affected by external factors. These infants have a higher chance of developing a flora that reflects the NICU, due to the immaturity of their GIs and prolonged exposure to the NICU (37). Infants who have undergone surgical enterostomy placement often have multiple predisposing factors that can perturb the intestinal microbiome, including premature birth, history of bowel injury or perforation, antibiotic exposure, prolonged withholding of enteral feeds, and intestinal surgery (38). Infants requiring intensive care are usually nursed in high-sanitary incubators and have restricted breastmilk intake and limited contact with their mother's skin (3). In addition, infants undergoing surgery of the GI tract commonly require some period of fasting, and often the use of gastric acid suppressants (39). These factors can cause early life dysbiosis, involving a delayed and suboptimal colonization of the intestine, which has been associated with long-term health conditions in adulthood (40). Research on the effects of intestinal surgery in neonates on longer-term gut microbiome changes is rare. However, Younge et al. (27) reported an overall decline over time in bacterial diversity during their 9 weeks' study of premature infants with enterostomy. In pathological conditions such as NEC, the diversity of the intestinal flora is impaired, resulting in an obvious trend toward simplification of the flora (41). A study (5) in our series of studies on FICare demonstrated that FICare can improve the richness and diversity of the intestinal microbiome in premature infants who didn't undergo surgery, with the establishment of intestinal flora that was close to that of term infants in the NICU who were breastfed and did not undergo surgery. In our study, the types of intestinal flora in the two groups were significantly different from those of normal children (25). Further study is needed to determine whether there is a difference in the established intestinal flora between children who underwent surgery and normal children.

The diversity of the intestinal flora can be an indicator of health status. However, we also found that multiple samples from the same individual exhibited great differences in microbial components and community structure. Therefore, it is crucial to be aware that the microbiota composition in the period after birth can be highly variable within an individual, and there are also enormous inter-individual differences in the colonization dynamics regarding the infant intestine (2, 42). There are no standardized definitions of the composition of a “healthy” GI microbiota at different developmental stages, and little is known about the main factors that contribute to the establishment of the GI microbiota in early life (25). GI microbial composition and species abundance in infants are affected by many variables that can directly or indirectly perturb the microbial community throughout the growth periods. The geographical region, host genetic factors, hygiene level of healthcare providers, mix-feeding (breastmilk and formula milk), brands and content of formula milk consumed, and other inter-individual variables are likely to contribute to GI microbiota development (43). One study (44) found that both the preterm infant microbiome and metabolome were highly specific to each individual and not associated with the infants' health conditions, and that the gut microbiome of preterm infants, who tend to be frequently treated with antibiotics, is enriched in microbes that commonly dominate after antibiotic exposure. Therefore, an individualized approach will be important to disentangle the health consequences of preterm infants' microbiomes.

It is important to note that the changes that we observed in the microbiome occurred in the context of the premature infant gut. Premature infants are still undergoing rapid intestinal growth and development, as well as maturation of the immune system. In addition, the infants were recovering from major intestinal injury and undergoing functional adaptation after loss of bowel length. It is possible that FICare may have differential effects on the microbiome and host at different stages of development. Given that the microbiome can vary significantly between individuals despite being in similar environments, and that some effects may take longer to occur than the length of this study, a larger, multicenter, longer-term study is necessary to explore the differences between FICare and non-FICare infants and to further examine the influence of confounding variables on the microbiome.

Limitations of this study were: (1) The sample size was small. It is very difficult to make a conclusion from a trial with such a small sample size. However, this was a pilot study for a group of specific preterm infants, who had enterostomy due to NEC with or without FICare during their recovering stage. Unfortunately, FICare is so far not a routine practice in NICUs in China yet, making the sample size even smaller. (2) The exposure time of our study might be too short to have detectable changes at later age (3–4 weeks postnatal) when stool microbiome is slower to change. Further studies are planned to follow these infants for their gut microbiome changes till their 1year corrected age. (3) Non-random allocation of the patients. Due to the ethical considerations, we have no way to randomize the eligible patients into FICare or non-FICare group, instead, we had to allocated the patients into 2 groups mainly based on their parents' willingness and choices. (4) The negative results of this study. As the first research to explore the feasibility and meaning of FICare for gut microbiome of NEC infants with enterostomy, we consider our study be a good try with fairly good novelty.

## Conclusion

In conclusion, short-term FICare had no significant positive effect on the establishment of intestinal flora diversity in premature NEC infants with enterostomy. Future studies will be conducted to assess the potential of FICare to modify infants' growth and development and intestinal adaptation, which may increase our ability to improve infants' development by improving their gut flora. We speculate that FICare may have broad practical implications, and the intervention will be improved by continuously assessing feedback and conducting follow-up studies.

## Data Availability Statement

The datasets presented in this study can be found in online repositories. The names of the repository/repositories and accession number(s) can be found below: National Genomics Data Center, CRA004032.

## Ethics Statement

The studies involving human participants were reviewed and approved by the Medical Ethics Committee of Beijing Children's Hospital (Registration No. 2017-106). Written informed consent to participate in this study was provided by the participants' legal guardian/next of kin.

## Author Contributions

MY, JD, MH, and MJ made substantial contributions to the conception of the study. MY, JD, QY, and WD helped the acquisition and analysis or interpretation of data for the work. MY drafted the work. MH and MJ revised it critically. All authors agreed to be accountable for all aspects of the work in ensuring that questions related to the accuracy or integrity of any part of the work are appropriately investigated and resolved. This draft was commented on by MH and MJ. MY and JD edited and approved the final draft. All authors contributed to the article and approved the submitted version.

## Conflict of Interest

The authors declare that the research was conducted in the absence of any commercial or financial relationships that could be construed as a potential conflict of interest.

## Publisher's Note

All claims expressed in this article are solely those of the authors and do not necessarily represent those of their affiliated organizations, or those of the publisher, the editors and the reviewers. Any product that may be evaluated in this article, or claim that may be made by its manufacturer, is not guaranteed or endorsed by the publisher.
